# Preanalytical approaches to improve recovery of amyloid-β peptides from CSF as measured by immunological or mass spectrometry-based assays

**DOI:** 10.1186/s13195-018-0445-0

**Published:** 2018-11-28

**Authors:** Stephen P. Schauer, William R. Mylott, Moucun Yuan, Rand G. Jenkins, W. Rodney Mathews, Lee A. Honigberg, Kristin R. Wildsmith

**Affiliations:** 1 Division of Development Sciences, Department of OMNI Biomarker Development, Genentech, Inc., 1 DNA Way, South San Francisco, CA 94080 USA; 2 PPD® Laboratories, 2240 Dabney Road, Richmond, VA 23230 USA

**Keywords:** Alzheimer’s disease, Adsorption, Biomarkers, Cerebrospinal fluid, Amyloid-β, Preanalytical, Immunoassay, LC-MS, Multiple-reaction monitoring (MRM), Diagnosis

## Abstract

**Background:**

Amyloid-β 1–42 (Aβ_1–42_) peptide is a well-established cerebrospinal fluid (CSF) biomarker for Alzheimer’s disease (AD). Reduced levels of Aβ_1–42_ are indicative of AD, but significant variation in the absolute concentrations of this analyte has been described for both healthy and diseased populations. Preanalytical factors such as storage tube type are reported to impact Aβ recovery and quantification accuracy. Using complementary immunological and mass spectrometry-based approaches, we identified and characterized preanalytical factors that influence measured concentrations of CSF Aβ peptides in stored samples.

**Methods:**

CSF from healthy control subjects and patients with AD was aliquoted into polypropylene tubes at volumes of 0.1 ml and 0.5 ml. CSF Aβ_1–42_ concentrations were initially measured by immunoassay; subsequent determinations of CSF Aβ_1–42_, Aβ_1–40_, Aβ_1–38_, Aβ_1–37_, and Aβ_1–34_ concentrations were made with an absolute quantitative mass spectrometry assay. In a second study, CSF from healthy control subjects and patients with dementia was denatured with guanidine hydrochloride (GuHCl) at different stages of the CSF collection and aliquoting process and then measured with the mass spectrometry assay.

**Results:**

Two distinct immunoassays demonstrated that CSF Aβ_1–42_ concentrations measured from 0.5-ml aliquots were higher than those from 0.1-ml aliquots. Tween-20 surfactant supplementation increased Aβ_1–42_ recovery but did not effectively resolve measured concentration differences associated with aliquot size. A CSF Aβ peptide mass spectrometry assay confirmed that Aβ peptide recovery was linked to sample volume. Unlike the immunoassay experiments, measured differences were consistently eliminated when aliquots were denatured in the original sample tube. Recovery from a panel of low-retention polypropylene tubes was assessed, and 1.5-ml Eppendorf LoBind® tubes were determined to be the least absorptive for Aβ_1–42_. A comparison of CSF collection and processing methods suggested that Aβ peptide recovery was improved by denaturing CSF earlier in the collection/aliquoting process and that the Aβ_1–42_/Aβ_1–40_ ratio was a useful method to reduce variability.

**Conclusions:**

Analyte loss due to nonspecific sample tube adsorption is a significant preanalytical factor that can compromise the accuracy of CSF Aβ_1–42_ measurements. Sample denaturation during aliquoting increases recovery of Aβ peptides and improves measurement accuracy. The Aβ_1–42_/Aβ_1–40_ ratio can overcome some of the quantitative variability precipitated by preanalytical factors affecting recovery.

**Electronic supplementary material:**

The online version of this article (10.1186/s13195-018-0445-0) contains supplementary material, which is available to authorized users.

## Background

Alzheimer’s disease (AD) is a chronic, progressive, and ultimately fatal neurodegenerative disease that accounts for 60–80% of clinical dementia cases [1]. AD is considered one of the largest unmet needs in medicine today, and intense efforts are underway to develop effective disease-modifying therapeutics. Amyloid-β 1–42 (Aβ_1–42_) is a well-characterized biomarker for AD [2]. This peptide can be detected in the cerebrospinal fluid (CSF), and concentrations are approximately twofold lower in patients with AD than in cognitively normal, age-matched control subjects [3]. Evidence suggests that changes in CSF Aβ_1–42_ concentrations precede amyloid deposition and inversely correlate with brain amyloid load [4–6].

Substantial variability in Aβ_1–42_ measurements has been reported in CSF biomarker studies [3, 7]. The peptide is exceedingly hydrophobic and therefore susceptible to adsorption and aggregation-related artifacts that can occur during preanalytical and analytical procedures [8–19]. Variability has also been linked to biological factors such as age, genetic background, and potential diurnal variation, which prompted changes to collection protocols, such as specifying a consistent time of day for collection [20–22]. Recent advances using automated platforms and reference calibrators have substantially reduced analytical sources of variability [23, 24], but efforts to identify and neutralize preanalytical factors that contribute to quantitative inconsistency are ongoing [17, 25].

In this study, we replicated the findings of Toombs et al. [16, 26], Berge et al. [27], and Vanderstichele et al. [28] by providing evidence that the combination of stored sample volume and tube surface adsorption is responsible for substantial variation in CSF Aβ_1–42_ peptide measurements. We reproduce findings that the preanalytical treatment of CSF with the detergent Tween-20 increases measured Aβ_1–42_ peptide from storage tubes and that Aβ_1–42_ recovery from low-binding tubes (Eppendorf Protein LoBind®; Eppendorf, Hauppauge, NY, USA) may be superior to that from other polypropylene tubes. We extended this work by demonstrating that Tween-20 increases Aβ_1–42_ measurements by facilitating recovery of tube-bound Aβ_1–42_, but we also show that it fails to consistently correct for sample volume-associated differences in quantitation driven by adsorption. Denaturation with guanidine hydrochloride (GuHCl) in quantitative mass spectrometry assay, however, does consistently improve recovery of Aβ_1–42_ as well as Aβ_1–40_, Aβ_1–38_, and Aβ_1–37_ while eliminating volume-associated inconsistencies. This assay also enabled us to explore the extent to which hydrophobicity impacts recovery of different forms of Aβ and to better understand how the use of a ratio of Aβ_1–42_ to Aβ_1–40_ may improve the quality and accuracy of CSF Aβ biomarker data.

## Methods

### CSF collection, aliquoting, and storage protocol used to evaluate the effect of aliquot volume and tube type on Aβ_1–42_ recovery

CSF samples were collected from young (ages 18–45 years), cognitively normal control subjects (YNC) and from patients clinically diagnosed with mild to moderate AD and confirmed to be amyloid-positive on the basis of a visual assessment of a florbetapir positron emission tomography (PET) scan by a central reader (Table 1). Samples were obtained from donors at 21 different collection sites, and the respective institutional review board approved the human collection protocols. All donors provided informed consent prior to the collection and use of their samples. CSF (10–12 ml) was collected by lumbar puncture between L4 and L5 with a Sprotte atraumatic needle, collected into 15-ml low-retention polypropylene tubes (62.554.205; Sarstedt AG, Numbrecht, Germany), frozen immediately on dry ice, transferred to a − 80 °C freezer for an average of 30 days, and then aliquoted on ice. In preparation for aliquoting, CSF was thawed on ice, vortexed for 30 s at maximum speed, and then centrifuged at 2000 × *g* for 3 min. Aliquots of 0.1 ml or 0.5 ml were dispensed into 0.5-ml low-retention screw-cap MAXYMum Recovery™ tubes (SCT-050-SS-L-C; Axygen Scientific Inc., Union City, CA, USA) (Additional file 1: Table S1) using MAXYMum Recovery™ low-retention pipette tips (T-1000-C-L; Axygen Scientific Inc.) and then frozen at − 80 °C.

**Table 1:** Demographics of cerebrospinal fluid donors

	CSF collection, aliquoting, and storage protocol used to evaluate effect of aliquot volume and tube type on Aβ_1–42_ recovery		Comparison of CSF collection, aliquoting, and storage protocols used to evaluate effect of GuHCl timing on Aβ recovery	
	YNC	AD	YNC	DEM
No. of subjects	20	82	10	10
Age, years, mean (SD)	36 (5.9)	72 (8.4)	30 (10)	68 (6.3)
Male, *n* (%)	11 (55%)	33 (40%)	6 (60%)	5 (50%)
Female, *n* (%)	9 (45%)	49 (60%)	4 (40%)	5 (50%)
MMSE score (SD)	NA	21 (0.80)	NA	20 (2.2)

*Abbreviations: Aβ* Amyloid-β, *AD* Alzheimer’s disease, *CSF* Cerebrospinal fluid, *DEM* Dementia of unknown origin, *GuHCl* Guanidine hydrochloride, *MMSE* Mini Mental State Examination, *YNC* Young normal control subjects

### Comparison of CSF collection, aliquoting, and storage protocols used to evaluate the effect of GuHCl timing on Aβ recovery

CSF (10–12 ml) was collected from ten YNC donors and ten patients clinically diagnosed with dementia (Table 1). CSF was collected as described above, with the following exceptions. The CSF was separated into 3 × 15-ml low-retention polypropylene tubes (62.554.205; Sarstedt AG) (Additional file 1: Table S1) immediately after collection (3 ml each), with one tube already containing 3 ml of 6 M GuHCl (condition 1). The 15-ml tubes were then frozen at − 80 °C. The remaining CSF (~ 1 ml) was aliquoted into 2 × 1.5-ml-capacity LoBind® tubes (0.5 ml each) (condition 4) (022431081; Eppendorf) (Additional file 1: Table S1) and then frozen at − 80 °C. After 24 h, the 15-ml tubes of CSF were thawed. The CSF that received GuHCl immediately after collection (condition 1) was aliquoted into 1.5-ml-capacity LoBind® tubes (0.5-ml each) and then frozen at − 80 °C. GuHCl (6 M, 3 ml) was added to one of the untreated tubes. The CSF was aliquoted into 1.5-ml-capacity LoBind® tubes (0.5 ml each) and then frozen at − 80 °C (condition 2). Untreated CSF in the last 15-ml conical tube was aliquoted into 1.5-ml-capacity LoBind® tubes (0.25 ml each) and then frozen at − 80 °C (condition 3). Aliquots from all conditions were thawed immediately before analysis, and the untreated aliquots (condition 3) then received 6 M GuHCl (0.25 ml each). A schematic of the conditions is shown in Fig. 7a. The two-dimensional ultraperformance liquid chromatography-tandem mass spectrometry (2D-UPLC-MS/MS) assay was performed as described above. Measurements were made within 2 weeks of collection.

### Luminex® xMAP multiplex immunoassay (AlzBio3)

Undiluted CSF (75 μl per determination) was mixed with polystyrene microspheres conjugated to three monoclonal capture antibodies (mAb) specific for Aβ_1–42_ (mAb 4D7A3, amino acids [aa] 37–42, GGVVIA), Tau (mAb AT120, aa 218–224, PPTREPK), p-Tau181 (mAb AT270, aa 175–181, PPAPKTP), and a solution of biotinylated detection antibodies against Aβ_1–42_ (mAb 3D6, aa 1–6, DAEFRH) and Tau (mAb HT7, aa 159–163, PPGQK) in a homogeneous sandwich immunoassay format. The intra- and interassay coefficients of variation (CVs) for this assay are < 20% [29] with an accuracy of > 80% (data not shown). After 18 h, the beads were washed with a phosphate buffer solution and then mixed with a solution of streptavidin-phycoerythrin for 1 h. Following a second wash with phosphate buffer, bead-bound analytes were quantified using a Bioplex® 200 multiplex array system (Bio-Rad Laboratories, Hercules, CA, USA). Bioplex Manager™ 6.1 software (Bio-Rad Laboratories) was used to fit six standards with a five-parameter logistic regression curve and interpolate sample concentrations. All determinations were made in duplicate. The lower and upper limits of quantification were defined as the lowest and highest calibrators whose technical replicates had a CV < 20% (Aβ_1–42_, 54 pg/ml and 1796 pg/ml; Tau, 25 pg/ml and 1554 pg/ml; phosphorylated Tau181 [pTau181], 15 pg/ml and 258 pg/ml).

### Meso Scale Discovery® Human Aβ_1–42_ Assay (MSD)

CSF Aβ_1–42_ concentrations were quantified with the Meso Scale Discovery® MULTI-SPOT® Human Aβ_1–42_ assay (K151LBE; Meso Scale Discovery, Gaithersburg, MD, USA), a solid-phase electrochemiluminescence (ECL) sandwich immunoassay specific for Aβ_1–42_. According to the manufacturer’s documentation, the intra- and interassay CVs for this assay are < 15% with an accuracy of 98%. CSF (10 μl) was removed from the original sample tubes with protein low-binding tips (2769-50; Thermo Fisher Scientific, Waltham, MA, USA) and was diluted 1:8 with 70 μl of assay diluent in 0.6 ml MAXYMum Recovery™ low-retention tubes (MCT-060-L-C; Axygen Scientific Inc.). Plates were blocked with assay diluent for 1 h and washed with PBS containing 0.2% Tween-20, then CSF and calibrators were added to each well where Aβ_1–42_ peptides were captured with an antibody specific for Aβ_1–42_ (mAb 12F4, aa 36–42, VGGVVIA) that was immobilized to the plate. After 1 h, the unbound analyte was washed away, and a detection antibody (mAb 6E10, aa 3-8, EFRHDS) that recognizes the N-terminus of Aβ peptides was added to each well. After 1 h, wells were washed again, an ECL reagent was added, and then the ECL intensity was quantified. Aβ_1–42_ concentrations were determined by interpolation from a seven-point, four-parameter logistic regression curve. All determinations were made in duplicate. The lower and upper limits of quantification for the assay were 3 pg/ml and 2000 pg/ml, respectively.

### 2D-UPLC-MS/MS

A 20-μl aliquot of 10 ng/ml internal standard working solution containing stable isotope-labeled internal standard Aβ peptides was added to 100-μl samples of human CSF or analyte-fortified artificial CSF (aCSF) containing buffers and electrolytes specified in the ALZET® formulation (150 mM Na^+^, 3.0 mM K^+^, 1.4 mM Ca^+ 2^, 0.8 mM Mg^+ 2^, 1.0 mM PO_4_^3−^, 155 mM Cl^−^; DURECT Corporation, Cupertino, CA, USA), 4 mg/ml human serum albumin, and 50 μg/ml immunoglobulin G. The samples were then mixed with 150 μl of 6 M GuHCl, incubated at 37 °C for 75 min, and then diluted with 200 μl of 4% H_3_PO_4_. Analytes were isolated from the pretreated CSF sample using an Oasis® MCX strong cation exchange mixed-mode μElution 96-well plate (Waters Corporation, Milford, MA, USA). After sequential washes with 4% H_3_PO_4_ and 10% acetonitrile (ACN), the analyte peptides were eluted in a small volume of ACN/water/NH_4_OH (75:15:10 vol/vol/vol). The extract was then diluted to 37.5% ACN with water. The relatively high organic and ammonium hydroxide composition of the extract was optimized to maintain solubility of the peptide analytes and minimize carryover effects.

The analytes were separated under high pH, NH_4_OH-based, reversed-phase liquid chromatographic conditions. Because the analytes vary in hydrophobicity and a large volume of relatively high organic extract was injected (30 μl), the technique of at-column dilution was used to ensure that the peptide analyte bands were efficiently focused on the precolumn. The solvent stream (comprised of 40% mobile phase B1 (ACN/methanol/trifluoroethanol 75:25:5 vol/vol/vol) carrying the injected sample plug from the autosampler was diluted fourfold with 0.3% aqueous ammonium hydroxide (to a weaker ~ 10% organic level) just prior to entering the trapping column (XBridge BEH C8 XP column, 2.1 × 30 mm, 2.5 μm; Waters Corporation). The focused analyte band was then back-transferred from the head of the precolumn onto the analytical column (Acquity UPLC® BEH300 C18, 2.1 × 150 mm, 1.7 μm; Waters Corporation), and the separation was carried out at 50 °C using a 3-min gradient from 20% to 45% mobile phase B2 (ACN/water/trifluoroethanol 90:5:5 vol/vol/vol). The eluted analyte peaks were detected by multiple-reaction monitoring (MRM) in positive electrospray ionization mode using a Xevo TQ-S mass spectrometer (Waters Corporation). The source temperature was 150 °C, the desolvation temperature was 600 °C, and the capillary voltage was 3.5 kV. MRM transitions, cone voltages, and collision energies for each peptide and internal standard are summarized in Additional file 1: Table S1. MassLynx instrument control software (Waters Corporation) was used for data acquisition. TargetLynx software (Waters Corporation) was used to integrate peak areas, perform regression analysis, and quantify the analytes. The data system was configured to automatically calculate and annotate the areas of the Aβ_1–34_, Aβ_1–37_, Aβ_1–38_, Aβ_1–40_, Aβ_1–42_, and the internal standard peaks. A calibration curve was constructed with the peak area ratios of the calibration standards by applying a linear, 1/concentration squared weighted, least-squares regression algorithm, and then all concentrations were calculated against the calibration line. The lower and upper limits of quantitation were 50 pg/ml and 5000 pg/ml for Aβ_1–34_, Aβ_1–37_, Aβ_1–38_, and Aβ_1–42_. The lower and upper limits of quantification for Aβ_1–40_ were 100 pg/ml and 10,000 pg/ml.

### Statistical analysis

Paired two-tailed *t* tests were used to compare Aβ_1–42_ concentrations recovered from matched 0.1-ml and 0.5-ml aliquots of CSF. Figures were generated with Spotfire® version 4.0.2 (TIBCO® Software Inc., Palo Alto, CA, USA) and Prism 6.0e (GraphPad Software, La Jolla, CA, USA) software.

### Data analysis for comparison of CSF collection, aliquoting, and storage protocols

Aβ concentrations recovered in condition 2, 3, or 4 were expressed as a percentage of Aβ concentrations recovered in condition 1 because the amount of Aβ recovered in condition 1 was the highest of the four conditions (Fig. 7a). The data were normalized according to the following formulas (Fig. 7b–d):

$$ \%\mathrm{of}\ \mathrm{A}\upbeta\ \mathrm{relative}\ \mathrm{to}\ \mathrm{Condition}\ 1=\left(\frac{{\left[\mathrm{A}\upbeta \right]}_{Condition\ 2,3, or\ 4}\ }{{\left[\mathrm{A}\upbeta \right]}_{Condition\ 1}}\right)\times 100 $$

Intrasubject percentage CV was calculated according to the following formula (Additional file 1: Figure S5):

$$ \mathrm{Intra}-\mathrm{Subject}\%\mathrm{CV}=\left(\frac{\mathrm{Standard}\ \mathrm{Deviation}\ \left(\mathrm{Mean}\ {\left[\mathrm{A}\upbeta 1-42\right]}_{Conditions\ 1,2,3,4}\ \right)}{\mathrm{Mean}\ {\left[\mathrm{A}\upbeta 1-42\right]}_{Conditions\ 1,2,3,4}}\right)\times 100 $$

Paired two-tailed *t* tests were used to compare normalized CSF Aβ_1–42_ recovery and intrasubject %CV from matched 0.5-ml aliquots.

## Results

### Sample volume impacts CSF Aβ_1–42_ measurements

**Fig. 1:**
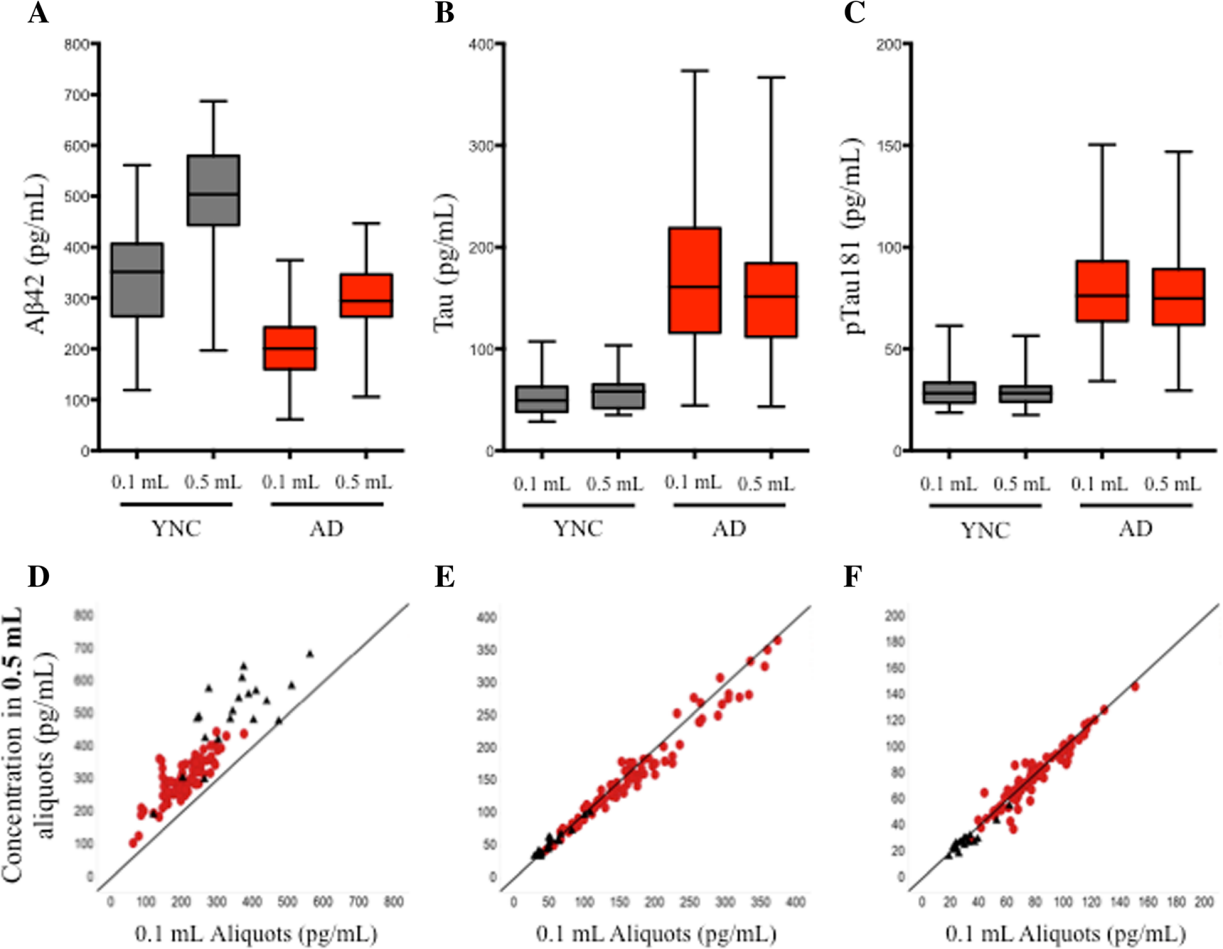
Impact of aliquot size on cerebrospinal fluid (CSF) amyloid-β 1–42 (Aβ_1–42_), Tau, and pTau181 concentrations measured from low-retention tubes. **a** CSF Aβ_1–42_ concentrations in 0.5-ml aliquots were higher than 0.1-ml aliquots when measured with the AlzBio3 assay. **b** and **c** Aliquot size did not have an appreciable impact on Tau and pTau181 concentrations. **d** CSF Aβ_1–42_ concentrations were biased toward higher concentrations in 0.5-ml aliquots (*r*_s_ = 0.84, *p* = 0.002). **e** CSF Tau concentrations were similar between 0.1-ml and 0.5-ml aliquots (*r*_s_ = 0.99, *p* < 0.0001). **f** CSF pTau181 concentrations were similar between 0.1-ml and 0.5-ml aliquots (*r*_s_ = 0.97, *p* < 0.0001). Young normal control subjects = black, triangles, *n* = 20; subjects with Alzheimer’s disease = red, circles, *n* = 81; solid line = line of identity (*y* = *x*); *r*_s_ = Spearman’s rank-correlation coefficient

CSF was collected from YNC and amyloid-PET-positive patients with AD. Aβ_1–42_, Tau, and pTau181 concentrations were measured from matched 0.1-ml and 0.5-ml aliquots using the AlzBio3 multiplexed immunoassay. Measured Aβ_1–42_ concentrations in 0.5-ml aliquots were higher than those obtained from 0.1-ml aliquots in both YNC (mean percentage increase = 50.5%, *p* < 0.0001) and patients with AD (mean percentage increase = 52.4%, *p* < 0.0001) (Fig. 1a, Table 2), raising the possibility that sample volume impacts Aβ_1–42_ measurements. Correlation plots of Aβ_1–42_ concentrations obtained from 0.1-ml versus 0.5-ml aliquots demonstrated that although there was some variability between patients, 0.5-ml aliquots consistently overrecovered relative to 0.1-ml aliquots (*r*_s_ = 0.84, *p* = 0.002) (Fig. 1d). The degree of overrecovery in 0.5-ml aliquots relative to 0.1-ml aliquots varied substantially from patient to patient (YNC range, + 10.2 to + 306 pg/ml; AD range, − 155 to + 227 pg/ml), and there was no statistically significant relationship between measured Aβ_1–42_ concentrations at either 0.1 ml or 0.5 ml and the difference in measured Aβ_1–42_ concentrations between 0.5 ml and 0.1 ml (YNC Spearman’s *R* = − 0.18, *p* > 0.05; AD Spearman’s *R* = − 0.15, *p* > 0.05). There were no correlations between the Aβ_1–42_ measurements and age, gender, weight, apolipoprotein E genotype, or study site (data not shown). In contrast, Tau and pTau181 concentrations were not impacted by aliquot size (Fig. 1b, c, e, and f, Table 2).

**Table 2:** Recovery of amyloid-β 1–42, Tau, and pTau181 from 0.1-ml and 0.5-ml aliquots

	Aβ_1–42_		Tau		pTau181	
	YNC	AD	YNC	AD	YNC	AD
No. of subjects	20	81	20	81	20	81
Mean concentration 0.1 ml (pg/ml)	344	204	55.1	174	30.5	78.2
Mean concentration 0.5 ml (pg/ml)	499	299	58.0	164	29.2	76.8
*p* Value (mean concentration 0.1 ml vs. 0.5 ml)	< 0.0001	< 0.0001	ns	< 0.0001	ns	0.021
Mean change from 0.1 ml to 0.5 ml (%)	50.5	52.4	7.59	− 4.77	− 2.42	− 1.74

*Abbreviations: Aβ* Amyloid-β, *AD* Alzheimer’s disease, *ns* Not significant, *YNC* Young normal control subject

*p* Values calculated by paired *t* test

To confirm that the relationship between sample volume and Aβ_1–42_ recovery was not an artifact of the AlzBio3 immunoassay, a subset of 0.1-ml and 0.5-ml aliquots from the AD group (*n* = 15) were assayed with the Meso Scale Discovery® human Aβ_1–42_ assay (MSD), a plate-based ECL immunoassay specific for Aβ_1–42_. In this subset, the mean AlzBio3-measured Aβ_1–42_ concentration in 0.5-ml aliquots was 61.5% greater than the mean concentration measured in 0.1-ml aliquots (*p* < 0.0001) (data not shown). In the MSD assay, the mean Aβ_1–42_ concentration measured from 0.5-ml aliquots in this subset was 108% greater than the mean concentration measured in matched 0.1-ml aliquots (*p* < 0.0001) (Additional file 1: Figure S1).

### Tween-20 recovers tube-bound Aβ_1–42_

To determine if the observed sample volume-associated differences in measured Aβ_1–42_ concentrations could be a function of tube adsorption, CSF from three patients with AD, specific to this substudy, were assayed after a 5-min treatment with a polysorbate surfactant capable of disrupting hydrophobic interactions (Tween-20, 0.2%). Aliquots of 0.5 ml from each donor were analyzed in pairs; one aliquot was pretreated with Tween-20 and then sampled for analysis, and the second aliquot was left untreated and then sampled for analysis. After the initial sampling, the remaining CSF was removed, the empty tubes were washed with 0.5 ml of artificial CSF containing Tween-20, and then the washes were sampled for analysis. The CSF and washes were measured concurrently with the AlzBio3 immunoassay, and concentrations reported for samples containing Tween-20 were adjusted to account for the dilution caused by the addition of the reagent (+ 2%).

**Fig. 2:**
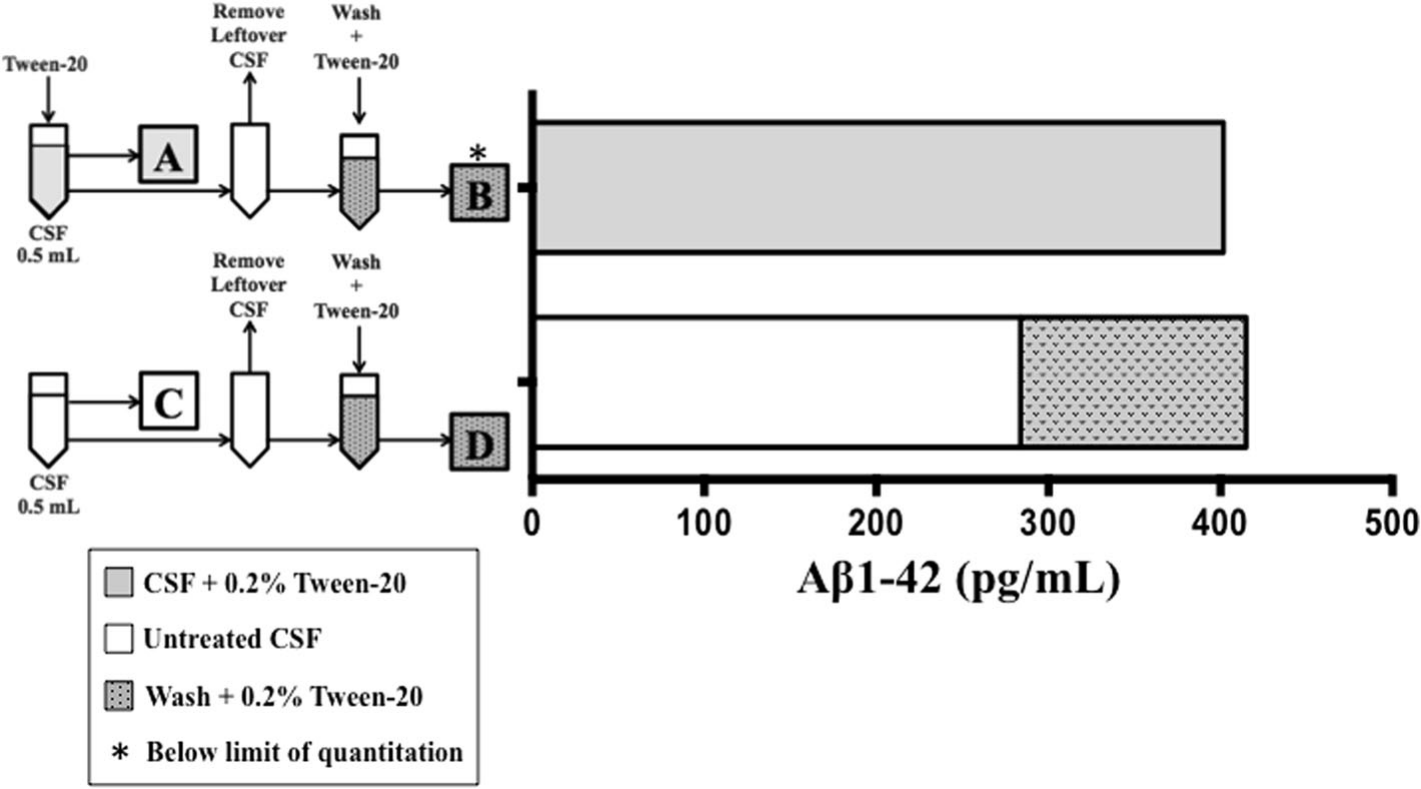
Tween-20 increased the recovery of cerebrospinal fluid (CSF) amyloid-β 1–42 (Aβ_1–42_) from low-retention polypropylene tubes as measured by the AlzBio3 assay. **a** Aβ_1–42_ recovery from 0.5-ml of CSF after Tween-20 pretreatment. **b** Aβ_1–42_ was not detected in artificial CSF + 0.2% Tween-20 tube wash. **c** Aβ_1–42_ recovery from 0.5-ml of CSF without Tween-20 pretreatment. **d** Aβ_1–42_ recovery from artificial CSF + 0.2% Tween-20 tube wash. Representative result, *n* = 1 patient with AD. White bars = untreated CSF, gray bars = CSF pretreated with 0.2% Tween-20, gray bars with dots = wash + 0.2% Tween-20. *Below limit of quantitation

The mean Aβ_1–42_ concentration between patient aliquots increased 53.1%, from 367 (±167) pg/ml to 561 (±249) pg/ml, when the CSF was pretreated with Tween-20. The peptide was undetectable in the washes obtained from the Tween-20-pretreated samples, but it was abundant in washes obtained from originally untreated aliquots (mean concentration = 216 [±126] pg/ml) (data not shown). Although these changes were not statistically significant when the patients were analyzed as a group, they were significant at the individual level. A representative result from one of the patients is shown in Fig. 2. The concentration of Aβ_1–42_ recovered from the Tween-20-treated aliquot (402 pg/ml) was 41.7% higher than that from the untreated aliquot (284 pg/ml) (*p* < 0.0001) (Fig. 2a and c). Aβ_1–42_ was not detected in the wash obtained from the Tween-20-treated aliquot (Fig. 2b), but a large quantity (131 pg/ml) was recovered in the wash obtained from the untreated aliquot (Fig. 2d). Interestingly, the sum of the Aβ_1–42_ recovered from the untreated aliquot and its subsequent wash (415 pg/ml) was almost equivalent to the amount of Aβ_1–42_ initially recovered from the aliquot pretreated with Tween-20 (402 pg/ml) (Fig. 2a versus Fig. 2c and d). In comparison, neither Tau nor pTau181 recovery was impacted by Tween-20 pretreatment, and neither analyte was detected in the wash (data not shown). These results suggest that a substantial quantity of CSF Aβ_1–42_ remains bound to polypropylene aliquot tubes, even when the tubes were specifically designed to minimize adsorption.

### Tween-20 does not resolve differences in CSF Aβ_1–42_ quantitation due to sample volume

**Fig. 3:**
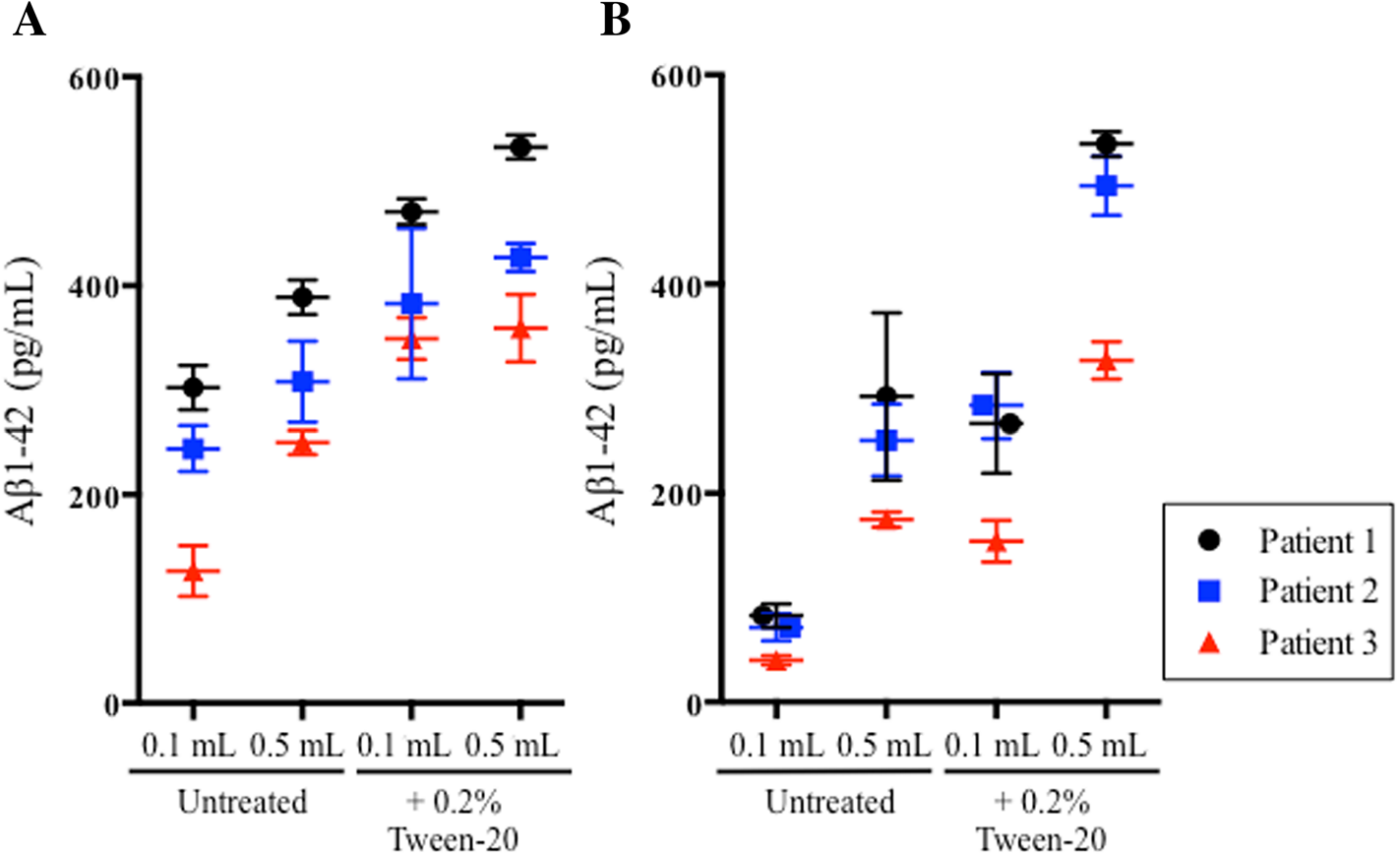
Tween-20 pretreatment increases recovery of cerebrospinal fluid (CSF) amyloid-β 1–42 (Aβ_1–42_) from 0.1-ml and 0.5-ml aliquots but fails to consistently normalize sample volume-associated difference between aliquots. **a** CSF Aβ_1–42_ concentrations from three patients with Alzheimer’s disease as measured by the AlzBio3 immunoassay. **b** CSF Aβ_1–42_ concentrations from the same patients, as measured by the MSD Aβ_1–42_ assay

Aliquots of 0.1 ml and 0.5 ml of CSF from patients with AD unique to this substudy (*n* = 3) were assayed on the AlzBio3 and MSD Aβ_1–42_ immunoassay platforms to determine if Tween-20 pretreatment would improve Aβ_1–42_ recovery and eliminate sample volume-associated quantitation differences. As measured by the AlzBio3 immunoassay, Tween-20 pretreatment increased the mean Aβ_1–42_ recovery in 0.1-ml and 0.5-ml aliquots by 96.2% (*p* < 0.0001) and 39.8% (*p* < 0.0001), respectively (Fig. 3a). Volume-associated differences in Aβ_1–42_ recovery were nearly eliminated after Tween-20 pretreatment in one of the patients and were reduced but not eliminated in the other two patients (Fig. 3a). When the same aliquots were measured with the MSD immunoassay, the mean Aβ_1–42_ recovery increased by 268% (*p* < 0.0001) in 0.1-ml aliquots and 88.9% (*p* < 0.0001) in 0.5-ml aliquots. Unlike the AlzBio3 immunoassay, however, there was no substantial reduction in volume-associated differences in Aβ_1–42_ recovery (Fig. 3b).

### Recovery of CSF Aβ_1–42_ from 1.5-ml Eppendorf LoBind® tubes is higher than that from other low-retention tube types

**Fig. 4:**
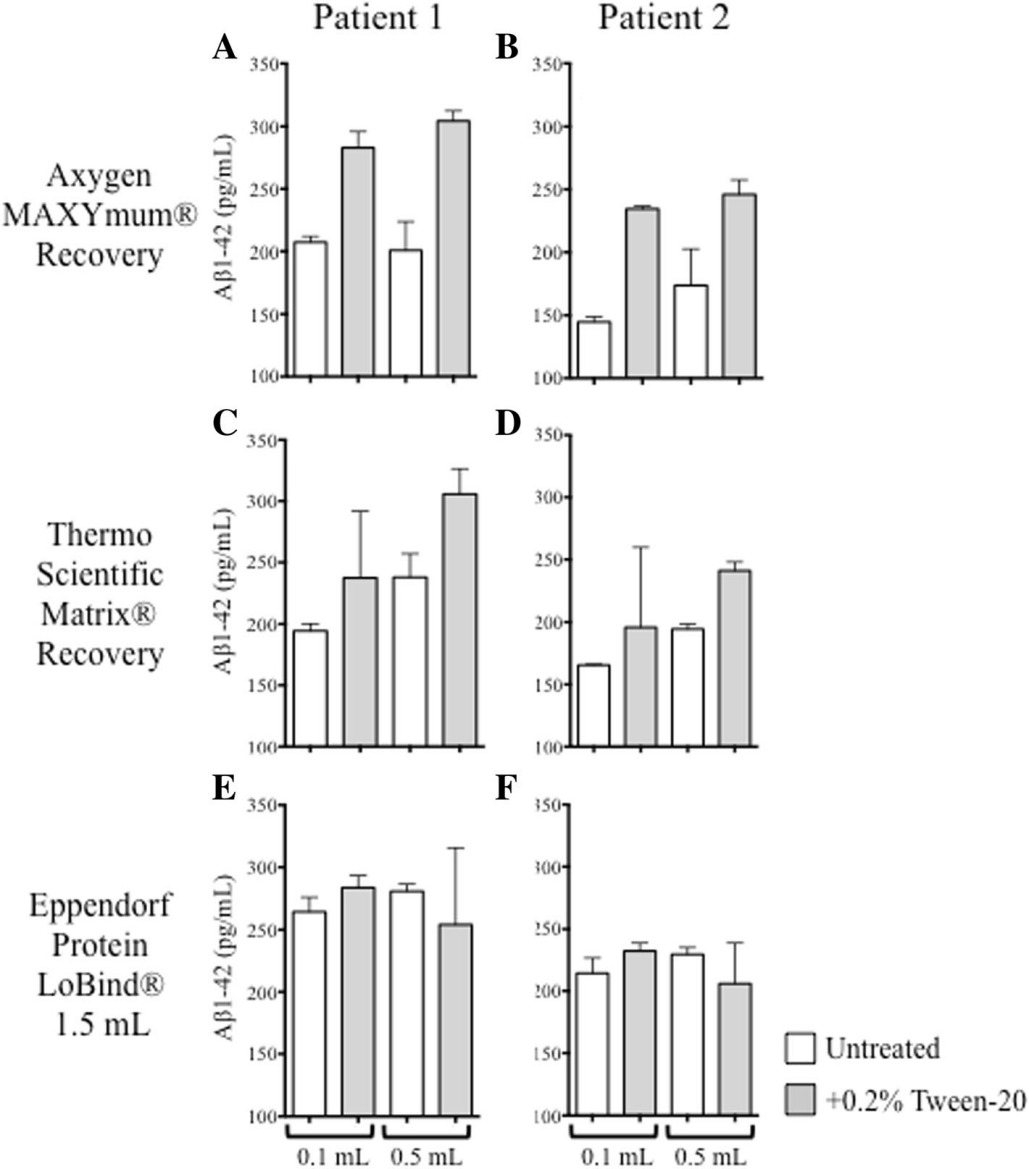
Amyloid-β 1–42 (Aβ_1–42_) concentrations recovered from 0.1-ml and 0.5-ml aliquots of cerebrospinal fluid (CSF) dispensed into different tube types with or without Tween-20 pretreatment as measured by the AlzBio3 assay. Aβ_1–42_ recovery from patients with Alzheimer’s disease (*n* = 2) after 0.1-ml or 0.5-ml volumes of CSF were dispensed into three low-retention polypropylene tube types and then assayed in the absence (white bars) or presence (gray bars) of Tween-20. **a** and **b** Axygen MAXYMum Recovery® tubes. **c** and **d** Thermo Scientific Matrix® tubes. **e** and **f** Eppendorf LoBind® tubes (1.5-ml capacity)

Because Aβ_1–42_ quantification is significantly impacted by tube adsorption, and because Tween-20 pretreatment failed to consistently normalize measurements between 0.1-ml and 0.5-ml aliquots, we examined Aβ_1–42_ recovery from two alternative low-retention tube types to determine whether preanalytical adsorption might be less confounding than what was observed in our original sample storage tubes. Collections of CSF (5 ml) were taken from two patients with AD unique to this substudy and were aliquoted into 1-ml-capacity Matrix® tubes (3740; Thermo Fisher Scientific), 1.5-ml-capacity LoBind® tubes, and MAXYMum® Recovery tubes at volumes of 0.1 ml and 0.5 ml. The aliquots were sampled in pairs, either untreated or after Tween-20 pretreatment. Measured Aβ_1–42_ concentrations from Matrix® tubes and MAXYMum® Recovery tubes increased significantly after Tween-20 pretreatment, demonstrating that Aβ_1–42_ adsorbed to these tubes (Fig. 4a–d). Juxtaposed to these results, Aβ_1–42_ recoveries from 1.5-ml LoBind® tubes were much higher in the absence of Tween-20; Tween-20 pretreatment did not facilitate additional peptide recovery, and more consistent results were yielded between 0.1-ml and 0.5-ml aliquots (Fig. 4e and f).

### Sample volume impact on CSF Aβ_1–42_ measurements confirmed with a 2D-UPLC-MS/MS assay

We sought an alternative assay method because Tween-20 pretreatment failed to consistently resolve discrepancies in Aβ_1–42_ quantitation by immunoassay. A clinical mass spectrometry assay (2D-UPLC-MS/MS) was identified that denatures Aβ in CSF with a strong chaotrope (GuHCl) and that was recently qualified for simultaneous measurement of five Aβ isoforms (Aβ_1–42_, Aβ_1–40_, Aβ_1–38_, Aβ_1–37_, and Aβ_1–34_) [30]. The multiplex nature of the assay also gave us the opportunity to test the link between Aβ hydrophobicity and decreasing peptide length; Aβ_1–42_ (DAEFRHDSGYEVHHQKLVFFAEDVGSNKGAIIGLMVGGVVIA), is the most hydrophobic isoform analyzed, and we hypothesized that the shorter isoforms would be less susceptible to preanalytical adsorption.

**Fig. 5:**
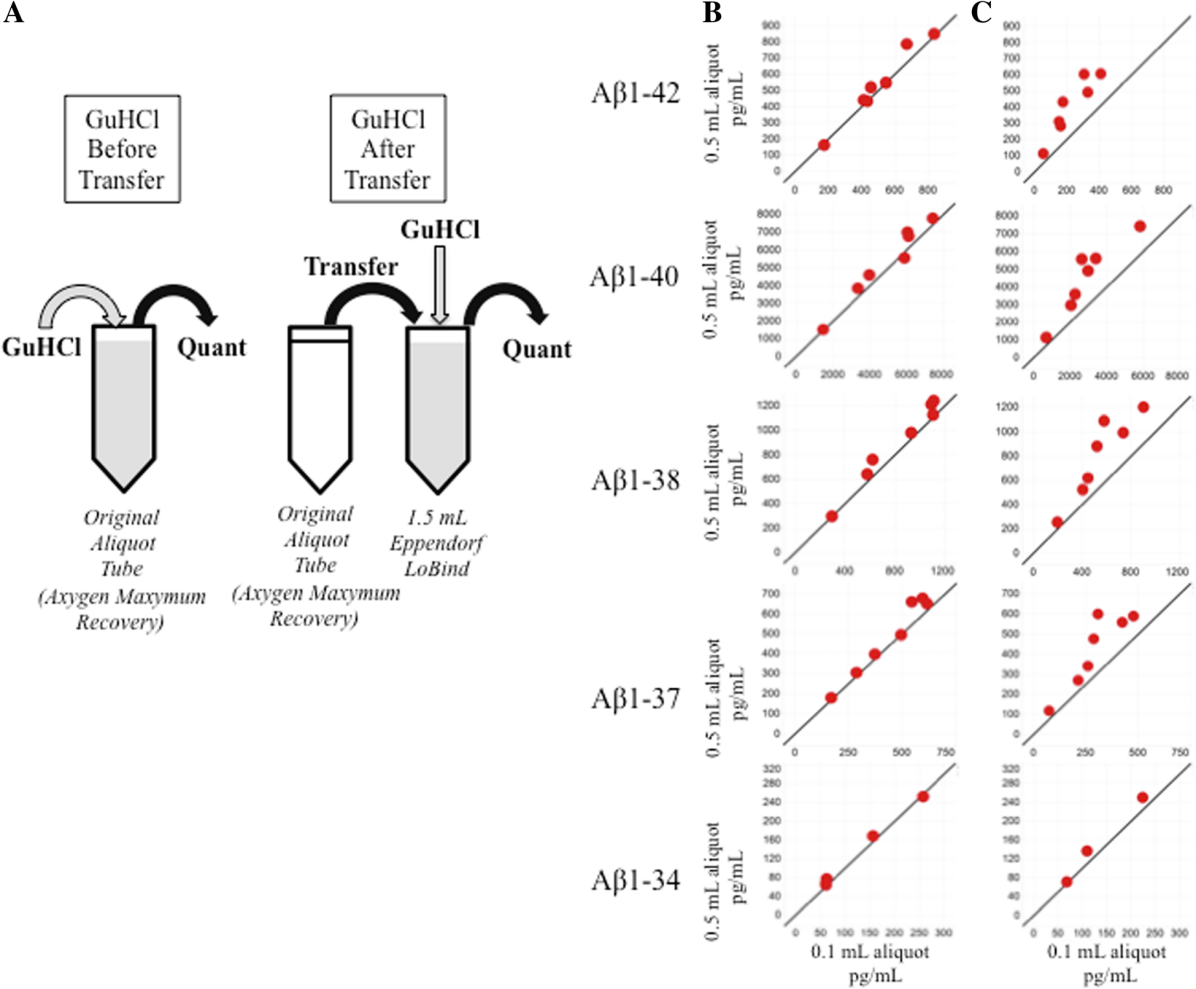
Guanidine hydrochloride (GuHCl) pretreatment increases cerebrospinal fluid (CSF) amyloid-β (Aβ) recovery and decreases aliquot size differences as measured by 2D ultraperformance LC-MS/MS. **a** Aliquots (0.1 ml and 0.5 ml) of CSF from patients with Alzheimer’s disease were denatured with GuHCl before the samples were removed from the aliquot tube (GuHCl Before Transfer) or after the samples were removed from the original aliquot tube (GuHCl After Transfer). **b** GuHCl denaturation before transfer increased Aβ peptide recovery while decreasing aliquot size-associated recovery differences (*n* = 7). **c** When CSF was denatured with GuHCl after the sample was transferred to another tube, Aβ peptide recovery was lower, and concentrations obtained from 0.5-ml aliquots were higher than those obtained from 0.1-ml aliquots (*n* = 7). Aβ_1–34_ concentrations were below the limit of detection for two and four subjects under each condition (**b** and **c**, respectively). Solid line = line of identity, where *y* = *x*

CSF aliquots from patients with AD (*n* = 7) unique to this substudy were selected. Following the qualified preanalytical assay workflow, a known volume of CSF was removed from the original aliquot and then transferred to a fresh tube containing an equal volume of denaturant (GuHCl). When 0.1-ml and 0.5-ml aliquots of AD CSF were processed by this method (GuHCl After Transfer, Fig. 5a), Aβ_1–42_ concentrations recovered from 0.5-ml aliquots were significantly higher than the concentrations recovered from 0.1-ml aliquots (*p* = 0.001) (Fig. 5c, Table 3). Aβ_1–40_, Aβ_1–38_, and Aβ_1–37_ concentrations also showed significant increases (*p* = 0.002, *p* = 0.0047, and 0.0074, respectively). Results for Aβ_1–34_ were inconclusive because concentrations were below the limit of detection for two and four subjects assayed under each condition (Fig. 5b and c, respectively; Table 3). We hypothesized if GuHCl was added directly to the original tube, it would solubilize and recover the entire quantity of Aβ present (GuHCl Before Transfer; Fig. 5a). When 0.1-ml and 0.5-ml aliquots of CSF from the same patients with AD were processed in this manner, the mean Aβ_1–42_, Aβ_1–40_, Aβ_1–38_, and Aβ_1–37_ recoveries increased relative to aliquots processed with the original sample preparation method (Fig. 5b). Notably, volume-associated Aβ_1–42_ recovery differences were eliminated when CSF was denatured in the original aliquot tube; the mean percentage difference in Aβ_1–42_ concentrations decreased from 90% (*p* = 0.001) with the original sample preparation method (GuHCl After Transfer; Fig. 5a) to 5% (*p* > 0.05) with the modified sample preparation protocol (GuHCl Before Transfer; Fig. 5a, Table 3). Significant reductions in volume-associated concentration differences were also observed for Aβ_1–40_, Aβ_1–38_, and Aβ_1–37_ (Table 3).

**Table 3:** GuHCl addition during sample preparation impacts amyloid-β peptide recovery from Alzheimer’s disease cerebrospinal fluid aliquots

	Aβ_1–42_		Aβ_1–40_		Aβ_1–38_		Aβ_1–37_		Aβ_1–34_	
	GuHCl before transfer	GuHCl after transfer	GuHCl before transfer	GuHCl after transfer	GuHCl before transfer	GuHCl after transfer	GuHCl before transfer	GuHCl after transfer	GuHCl before transfer	GuHCl after transfer
No. of subjects	7	7	7	7	7	7	7	7	5	3
Difference between 0.1-ml and 0.5-ml aliquots (%)	5	**90**	9	**63**	9	**46**	9	**48**	10	14
SD (%)	8	35	8	26	7	23	7	27	10	10
Range (%)	− 6 to 18	48–146	− 5 to 16	27–112	1–23	30–89	0–22	24–95	− 2 to 25	5–25
*p* Value (0.1 ml vs. 0.5 ml)	0.1103	0.001	0.043	0.002	0.0104	0.0047	0.0428	0.0074	0.1199	0.1368

*Abbreviations: Aβ* Amyloid-β, *AD* Alzheimer’s disease, *GuHCl* Guanidine hydrochloride

*p* values were calculated by paired *t* test. Boldface values indicate noteworthy changes concomitant with a *p* value < 0.05

Difference between 0.1-ml and 0.5-ml aliquots (%) = ([b − a]/[a]) × 100, where:

a = [Aβ_1-X_] recovered from 0.1-ml aliquots

b = [Aβ_1–X_] recovered from 0.5-ml aliquots

### GuHCl recovers tube-bound Aβ_1–42_ from healthy and AD CSF

To determine whether a GuHCl-facilitated increase in CSF Aβ_1–42_ recovery from aliquot tubes would impact the capacity of this biomarker to discriminate between YNC and patients with AD, sets of 0.1-ml CSF aliquots from YNC (*n* = 17) and patients with AD (*n* = 20) were prepared for analysis with each of the sample preparation workflows (GuHCl After Transfer, GuHCl Before Transfer; Fig. 5a) and then analyzed with the 2D-UPLC-MS/MS assay. When CSF was denatured with GuHCl in the original aliquot tube, the mean CSF Aβ_1–42_ recovery increased 86% (*p* < 0.0001) in YNC and 107% (*p* < 0.0001) in patients with AD relative to matched aliquots processed with the original sample preparation workflow (Additional file 1: Figure S2a and b). Significant increases in CSF Aβ_1–40_, Aβ_1–38_, and Aβ_1–37_ concentrations were also observed in both groups, although the magnitude of the change lessened as the length and hydrophobicity of the Aβ peptide decreased (data not shown). ROC curves were generated to determine whether the capacity of Aβ_1–42_ to discriminate between YNC and patients with AD was impacted by increased recovery. Although values increased in both YNC and AD when GuHCl was added directly to CSF aliquots, there was no significant difference in the diagnostic performance of this biomarker. The AUC was 0.8588 (95% CI, 0.7329, 0.9487) when GuHCl was added directly to CSF aliquots, and the AUC was 0.8453 (95% CI, 0.6980, 0.9727) when GuHCl was added after the CSF was transferred out of the original storage tube (Additional file 1: Figure S3).

### Recovery of CSF Aβ_1–42_ from 1.5-ml Eppendorf LoBind® tubes is higher than that from Axygen MAXYMum® Recovery tubes as measured by 2D-UPLC-MS/MS assay

**Fig. 6:**
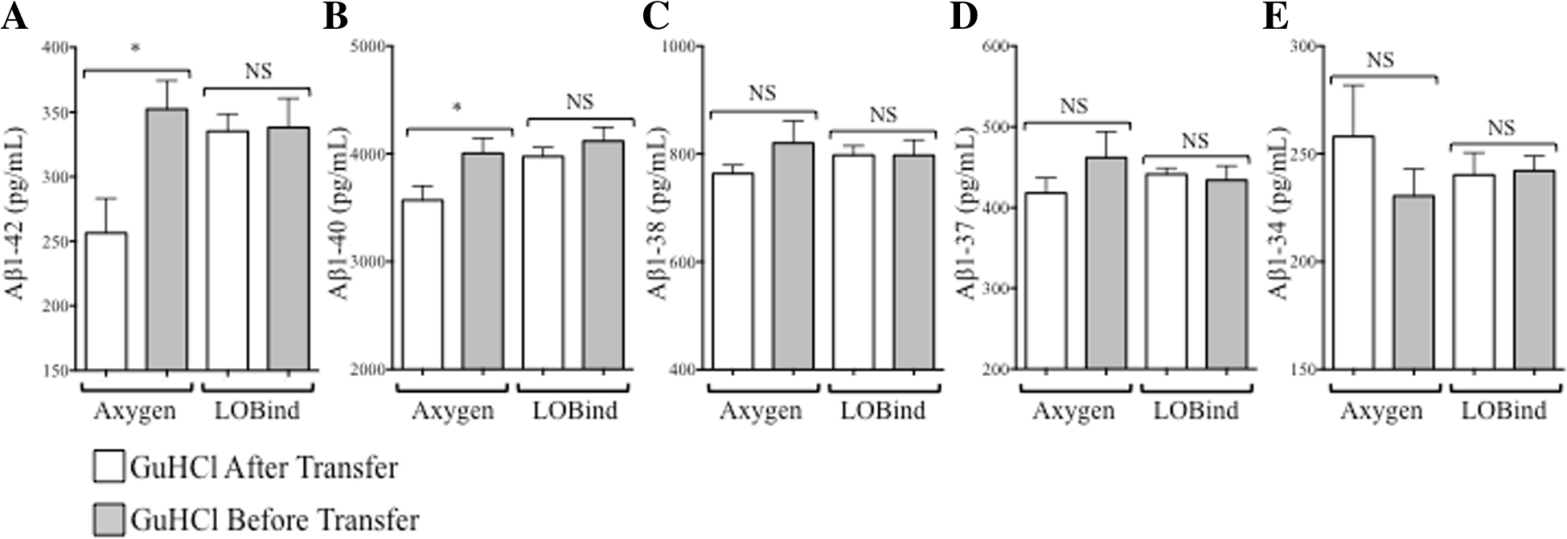
Amyloid-β (Aβ) peptide recovery from cerebrospinal fluid (CSF) aliquoted into Axygen MAXYMum Recovery® or Eppendorf LoBind® tubes following guanidine hydrochloride (GuHCl) denaturation before tube transfer (gray bars) or after transfer to a new tube (white bars) as measured by 2D ultraperformance LC-MS/MS. **a** and **b** Aβ_1–42_ and Aβ_1–40_ concentrations were significantly higher (*p* ≤ 0.05) when pooled Alzheimer’s disease CSF from four patients was denatured with GuHCl in the original Axygen MAXYMum Recovery® aliquot tubes before analysis (Axygen, gray bars) when compared with concentrations recovered from CSF denatured with GuHCl after removal from the original aliquot tube (white bars). Aβ_1–42_ and Aβ_1–40_ concentrations from the same CSF pool were higher when measured from Eppendorf LoBind® tubes, regardless of the timing of GuHCl treatment. **c**–**e** Aβ_1–38_, Aβ_1–37_, and Aβ_1–34_ concentrations were not significantly impacted by tube type or GuHCl pretreatment. *p ≤ 0.05, NS = not significant (*p* > 0.05)

Our immunoassay data suggested that Aβ_1–42_ recovery could be improved by using 1.5-ml Eppendorf LoBind® tubes, so we decided to test if this finding could be reproduced on the mass spectrometry assay platform. A pool of AD CSF was created and then dispensed into 0.5-ml Axygen MAXYMum® Recovery tubes or 1.5-ml Eppendorf LoBind® tubes with 0.1-ml volumes. The new aliquots were denatured with each preanalytical workflow (Fig. 5a) and then measured by 2D-UPLC-MS/MS. Consistent with the previous immunoassay experiments, Aβ_1–42_ recovery from 1.5-ml Eppendorf LoBind® tubes was higher than recovery from Axygen MAXYMum® Recovery tubes, regardless of the denaturation method (Fig. 6a). In comparison, Aβ_1–42_ recovery from 0.5-ml Axygen MAXYMum® Recovery tubes was dependent on how the sample was denatured; the recovery was significantly higher when CSF was denatured in the original tube (Fig. 6a). Similar results were also observed for CSF Aβ_1–40_ (Fig. 6b). Recovery of the less hydrophobic peptides (Aβ_1–38_, Aβ_1–37_, Aβ_1–34_) were not significantly impacted by tube type or GuHCl denaturation (Fig. 6c–e).

### Aβ_1–42_ recovery from Eppendorf LoBind® tubes improves when CSF is denatured earlier in collection and aliquoting process

**Fig. 7:**
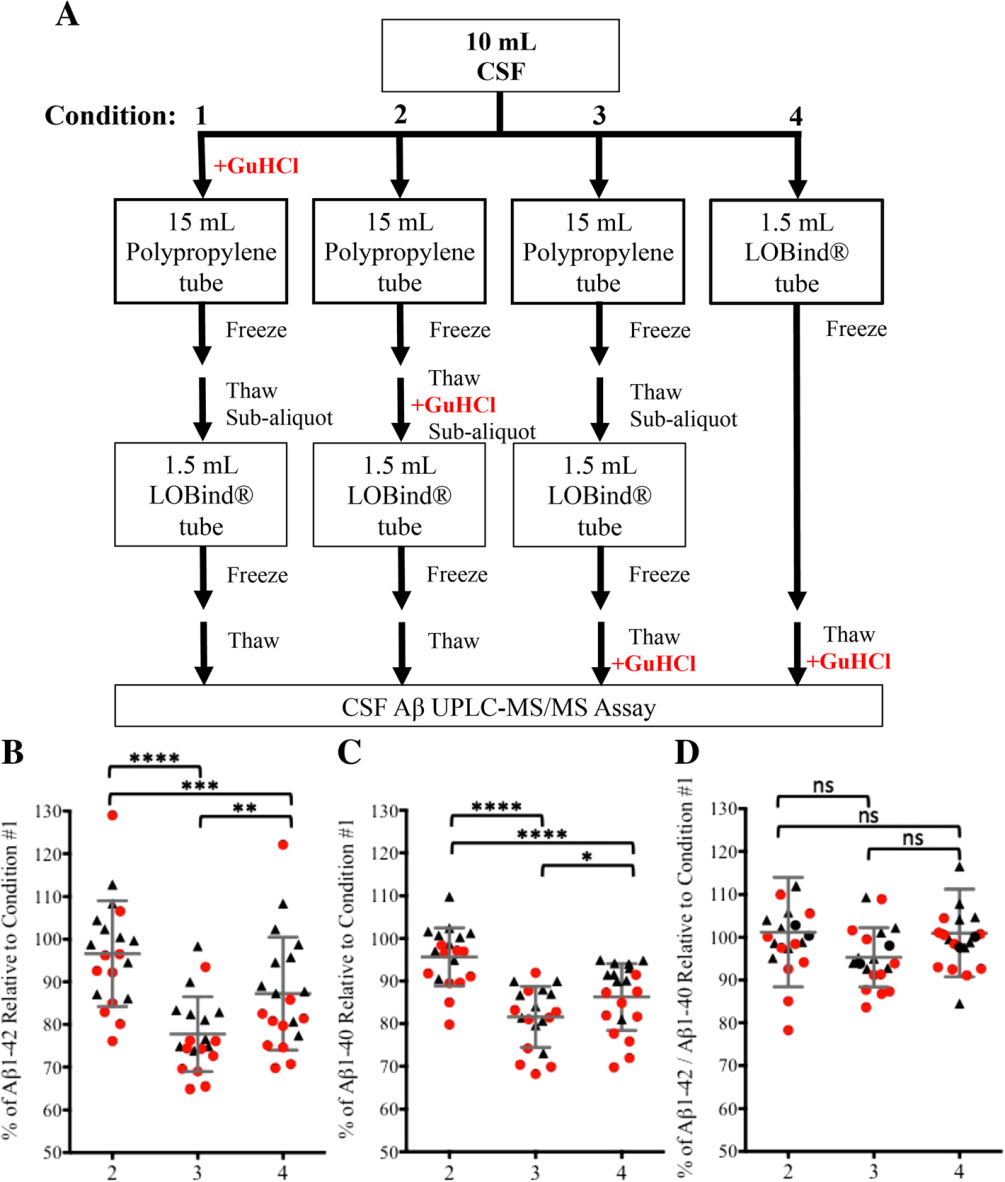
The timing of guanidine hydrochloride (GuHCl) denaturation impacts baseline cerebrospinal fluid (CSF) amyloid-β 1–42 (Aβ_1–42_) recovery in healthy control subjects and subjects with dementia as measured by 2D ultraperformance LC-MS/MS. **a** CSF collection workflows to assess the impact of earlier GuHCl denaturation on CSF Aβ recovery from Eppendorf LoBind® tubes. Condition 1: GuHCl added to CSF immediately after collection; condition 2: GuHCl denaturation immediately before subaliquoting; condition 3: GuHCl denaturation after subaliquoting and immediately before analysis; condition 4: into LoBind® tubes immediately after collection, GuHCl denaturation immediately before analysis. **b** Aβ_1–42_ concentrations recovered from the CSF of control subjects (black triangles, *n* = 10) and patients with dementia (red circles, n = 10) in conditions 2, 3, and 4 as a percentage of individual recoveries in condition 1. **c** Aβ_1–40_ concentrations recovered from the CSF of control subjects (black triangles) and patients with dementia (red circles) in conditions 2, 3, and 4 as a percentage of individual recoveries in condition 1. **d** CSF Aβ_1–42_/Aβ_1–40_ ratios from control subjects (black triangles) and patients with dementia (red circles) in conditions 2, 3, and 4 as a percentage of the ratio in condition 1. All measurements were made within 2 weeks of collection. ****p ≤ 0.0001, ***p ≤ 0.001, **p ≤ 0.01, *p ≤ 0.05, NS = not significant (*p* > 0.05)

In the case where we were able to prospectively collect CSF and store aliquots in Eppendorf LoBind® tubes, we wanted to determine whether Aβ_1–42_ recovery could be increased further by adding GuHCl to CSF at earlier stages of the collection and aliquoting process. CSF from healthy volunteers (*n* = 10) and patients diagnosed with dementia of unknown origin (*n* = 10) was collected into 15-ml polypropylene tubes. The CSF was immediately divided into three workflows: GuHCl denaturation in the 15-ml polypropylene tube immediately after collection (condition 1, Fig. 7a), GuHCl denaturation in the 15-ml polypropylene tube after one freeze-thaw cycle, immediately before subaliquoting into Eppendorf LoBind® tubes (condition 2, Fig. 7a), or GuHCl denaturation in Eppendorf LoBind® tubes after subaliquoting immediately before analysis (condition 3, Fig. 7a). A fourth workflow tested Aβ_1–42_ recovery after the CSF was aliquoted directly into 1.5-ml Eppendorf LoBind® tubes after collection, with GuHCl denaturation occurring immediately before analysis, after one freeze-thaw cycle (condition 4, Fig. 7a). All measurements were made within 2 weeks of collection.

Aβ_1–42_ recovery was highest when GuHCl denaturation occurred in 15-ml polypropylene collection tubes immediately after collection, regardless of the diagnostic group (condition 1, Additional file 1: Figure S4a and b), and thus this condition was used for reference. When GuHCl denaturation was delayed until immediately before the subaliquoting process, after the CSF had undergone a single freeze-thaw (condition 2, Fig. 7a), Aβ_1–42_ recovery decreased nonsignificantly (*p* > 0.05) relative to condition 1 (Fig. 7b, Additional file 1: Figure S4a and b). However, when GuHCl denaturation was postponed until after CSF had been aliquoted into Eppendorf LoBind® tubes, the mean Aβ_1–42_ concentration recovered from both diagnostic groups decreased by 22% relative to condition 1 (*p* < 0.0001) (Fig. 7b, Additional file 1: Figure S4a and b). When CSF was aliquoted directly into LoBind® tubes after collection (condition 4, Fig. 7a), recovery was 15% (*p* = 0.0008) less than condition 1, but 9% (*p* = 0.0025) higher than condition 3 (Fig. 7b, Additional file 1: Figure S4a and b). The timing of GuHCl denaturation had comparable effects on the magnitude and significance of Aβ_1–40_ recovery (Fig. 7c, Additional file 1: Figure S4c and d), whereas it had a smaller impact on Aβ_1–38_, Aβ_1–37_, and Aβ_1–34_ recoveries (Additional file 1: Figure S4e, f, g, h, i, j). Recovered Aβ_1–42_ concentrations were normalized to Aβ_1–40_ to determine if the ratio would be less variable than the absolute concentrations (Fig. 7d). An intrasubject, intercondition %CV was generated for the mean Aβ_1–42_ concentration and Aβ_1–42_/Aβ_1–40_ ratio across conditions 1–4. The intercondition %CV for Aβ_1–42_ was 13.5%, reducing significantly to 5.71% when the Aβ_1–42_/Aβ_1–40_ ratio was applied to the data (*p* < 0.0001) (Additional file 1: Figure S5). Similarly, the Aβ_1–42_/Aβ_1–38_ and Aβ_1–42_/Aβ_1–37_ ratios significantly (*p* < 0.0001) reduced differences in recovery between collection conditions (Additional file 1: Figure S5).

## Discussion

Rigorous biomarker development efforts have validated reduced CSF Aβ_1–42_ levels as diagnostic for AD [31], and recent clinical trials have demonstrated that the peptide is a useful pharmacodynamic biomarker for antiamyloid therapeutics [32]. In spite of these successes, the AD biomarker field has struggled with quantitative consistency, and significant variation in the absolute levels of CSF Aβ_1–42_ has been reported [33]. Although there have been substantial improvements in the accuracy, precision, and robustness of the assays designed to quantify CSF Aβ_1–42_ [23], the identification and elimination of preanalytical factors responsible for quantification irregularities is an ongoing effort. In this study, we identified tube adsorption and sample volume as modifiable preanalytical factors that combine to create substantial variability in CSF Aβ measurements.

In the first part of study, we observed significant differences in the amount of Aβ_1–42_ recovered from matched 0.1-ml and 0.5-ml aliquots of CSF measured with a Luminex® immunoassay (Luminex, Austin, TX, USA). In contrast, CSF Tau and pTau181 measurements were stable, regardless of sample volume, suggesting that the effect was specifically associated with the hydrophobicity of Aβ_1–42_ and its tendency to nonspecifically bind (adsorb) to polypropylene sample tubes [12–17, 26, 34]. This trend was confirmed with a second immunoassay (Meso Scale Discovery® ECL assay), but with a higher reported difference between concentrations recovered from the aliquots. This could be a consequence of a difference between the assay calibrators, calibration curve ranges, or detection methods used in the assays; alternatively, the MSD assay protocol entailed a CSF predilution, which could have potentially contributed to differences in overall reported concentrations relative to the Luminex® assay. Nonetheless, the results demonstrate that measured Aβ_1–42_ concentrations recovered from 0.5-ml aliquots are consistently higher than those from 0.1-ml aliquots, independent of immunoassay platform. We replicated this finding a third time by measuring a subset of 0.1-ml and 0.5-ml aliquots with an absolute quantitative 2D-UPLC-MS/MS assay. The MRM method used for these measurements was developed in association with the Global Biomarkers Standardization Consortium of the Alzheimer’s Association and quantifies five Aβ peptides simultaneously (Aβ_1–42_, Aβ_1–40_, Aβ_1–38_, Aβ_1–37_, Aβ_1–34_) [24, 25, 35, 36], providing a more comprehensive representation of the effects of amyloid precursor protein processing than Aβ_1–42_ alone, and it enabled us to observe whether different forms of Aβ may be less impacted by preanalytical factors. This effect declined progressively as the length and hydrophobicity of the measured peptide decreased, supporting the concept that tube adsorption is a function by hydrophobicity, and this finding is consistent with recently reported results using multiplex immunoassays [26].

To determine the extent to which tube adsorption was a factor in these observations, CSF aliquots were pretreated with Tween-20, a polysorbate surfactant capable of disrupting hydrophobic interactions. Aβ_1–42_ concentrations increased following pretreatment, similar to previous reports [13, 15, 16, 27, 28], and a tube wash experiment implied that a large portion of the available Aβ_1–42_ population was tube-bound and therefore unmeasured by the assay, not masked by matrix interference as reported by others [18, 19]. Had the peptide been masked by matrix interference or conformation, we would not have recovered Aβ_1–42_ from the walls of emptied tubes by washing with aCSF and Tween-20. Because Tween-20 had such a profound effect on recovery, we hypothesized that sample pretreatment might be an easy way to limit peptide adsorption and eliminate volume-associated quantitative discrepancies. In our hands, however, this led to inconsistent results, and we hypothesized that Tween-20 might be interfering with immunoassay components as reported by Vanderstichele et al. [28].

Knowing from our Tween-20 recovery experiments that Aβ_1–42_ adsorbed to the tubes, the original 2D-UPLC-MS/MS sample preparation workflow were modified to denature CSF in the original sample tube and maximize recovery by solubilizing tube-bound peptides. When matched pairs of 0.1-ml and 0.5-ml aliquots of AD CSF from the same donors were prepared in this manner, measured concentrations of Aβ_1–42_ increased, and sample volume-associated differences were eliminated. Similar results were also observed for Aβ_1–40_, Aβ_1–38_, and Aβ_1–37_. These results demonstrate that it is possible to eliminate inconsistencies in CSF Aβ_1–42_ quantification due to preanalytical tube adsorption with a simple modification to an existing sample preparation procedure, rather than introducing substances that could interfere with assay components.

There is a limitation to the approach of denaturing CSF in the original sample tube: Concentrations calculated using the 2D-UPLC-MS/MS assay are dependent on the accuracy of the sample volume processed for the method. In a routine clinical setting, it may be difficult to ensure that a precise and accurate volume of CSF is collected into the tube, which may limit the broad adoption of this approach. Because the LC-MS platform is routinely used for absolute quantitation, this approach could be effectively applied as a reference or comparator method. The multiplex LC-MS assay used in our work was developed in collaboration with the Global Biomarker Standardization Consortium of the Alzheimer’s Association and the International Federation of Clinical Chemistry and Laboratory Medicine Working Group on CSF Proteins. It was used during global interlaboratory studies conducted to develop and qualify candidate LC-MS-based reference methods and establish values for human CSF-certified reference materials produced by the European Commission Joint Research Centre to aid in harmonizing Aβ_1–42_ biomarker assays across all technologies [24, 25, 30, 35, 36]. The approach to denaturation in the original sample tube could also be applied when the method is used as a pharmacodynamic biomarker assay to assess the biological effects of therapeutics in development. However, the issue could be avoided if GuHCl denaturation in the original tube were made unnecessary. We addressed this question using 1.5-ml capacity Eppendorf LoBind® tubes, which were substantially less adsorptive than the other tube types tested in our immunoassay experiments. Aβ_1–42_ recovery measured by AlzBio3 from untreated CSF in 1.5-ml Eppendorf LoBind® tubes exceeded untreated recovery and approached or exceeded Tween-20-facilitated recovery from the other tube types. In the 2D-UPLC-MS/MS assay, regardless of whether CSF was denatured directly in the tube or transferred to a fresh tube containing denaturant, Aβ_1–42_ recovery from 1.5-ml Eppendorf LoBind® tubes exceeded recovery from polypropylene tubes. Both our immunoassay and 2D-UPLC-MS/MS results suggest that 1.5-ml-capacity Eppendorf LoBind® tubes be considered for the storage of CSF intended for Aβ_1–42_ analysis and are consistent with recommendations by Vanderstichele et al. [28].

Improved recovery could negatively impact the diagnostic power of CSF Aβ_1–42_. For example, if CSF Aβ_1–42_ peptides from patients with AD are more adsorptive than peptides obtained from healthy control subjects, differential tube retention might explain why measured concentrations differ between control subjects and patients with AD by as much as twofold [3]. However, denaturation in the original aliquot tube increased Aβ_1–42_ recovery in both patients with AD and control subjects, and ROC curve analysis did not indicate that there was an impact on the diagnostic power of the analyte, suggesting that the discriminatory power of Aβ_1–42_ is not dependent on tube adsorption.

With experimental evidence that Aβ peptide adsorption occurs in sample aliquots and additional published data demonstrating that it occurs within 30 s of exposure to an adsorbent [34], it is likely that the phenomenon also occurs during the CSF collection and processing steps upstream of aliquot creation. We evaluated the impact of earlier denaturation and found notable increases in Aβ_1–42_ recovery when denaturation was initiated sooner, with the highest recovered concentrations detected in samples denatured immediately after collection or immediately before subaliquoting. These results demonstrate that analyte loss also occurs during the collection and aliquoting process. From a clinical assay perspective, the option to delay GuHCl denaturation until subaliquoting provides valuable flexibility; rather than commit an entire CSF draw to a specific assay such as 2D-UPLC-MS/MS, an investigator can postpone GuHCl denaturation until the subaliquoting step without risking Aβ loss, creating samples both with and without denaturant, thereby allowing additional biomarker measurements on platforms that may not be GuHCl-compatible.

It has recently been reported that the CSF Aβ_1–42_/Aβ_1–40_ ratio strongly correlates with brain amyloid load and is considerably more effective at diagnosing clinical AD than CSF Aβ_1–42_ alone [28, 34, 37–42]. It is unclear whether the ratio may be a better diagnostic because it corrects for sample loss or because it reflects the shift in amyloid precursor protein metabolism that leads to the aggregation of Aβ_1–42_. These reports have generated substantial interest in the field of AD biomarkers, and the potential impact of this ratio continues to be explored. Beyond diagnostic applications, Aβ peptide ratios have also been applied to preanalytical questions and are reported to be useful in ameliorating the effects of inconsistent recovery due to adsorption, although there has been some inconsistency in the results [26, 34]. A strength of the 2D-UPLC-MS/MS Aβ peptide assay is its capacity to measure Aβ_1–42_ and Aβ_1–40_ concentrations from the same aliquot simultaneously, thereby eliminating intra-aliquot, interassay, and interplatform inconsistencies that could potentially confound the accuracy of the Aβ_1–42_/Aβ_1–40_ ratio. We were curious what impact normalization of Aβ_1–42_ to Aβ_1–40_ would have on the Aβ_1–42_ recovery differences that emerge based on the timing of denaturation. We found that normalization to Aβ_1–40_ substantially reduced measured variability in recovery for all subjects, as did normalization to Aβ_1–38_ and Aβ_1–37_. The Aβ_1–42_/Aβ_1–40_ ratio demonstrated the lowest variability, possibly owing to the higher abundance of Aβ_1–40_ in CSF, and of the isoforms evaluated, it was closest in hydrophobicity to Aβ_1–42_. These results suggest that it may be possible compensate for inconsistent absolute Aβ_1–42_ recoveries by performing a simple normalization step.

## Conclusions

This study confirms previous observations that CSF Aβ nonspecifically adsorbs to sample tubes, that disproportionate adsorption can occur as a function of sample volume, and that this can have a significant effect on measured peptide recovery. Interestingly, tube adsorption may not impact diagnostic performance of Aβ_1–42_, because improved recovery did not reduce the discrimination between patients with AD and control subjects. Maximum recovery of Aβ was observed when using 1.5-ml Eppendorf LoBind® tubes as assessed by immunoassay and a 2D-UPLC-MS/MS assay. Recovery can be further improved with denaturation of CSF using GuHCl early in the handling process. Collection of CSF directly into LoBind® tubes should also be explored as a strategy to resolve the adsorption issue. Additionally, our study further supports that the ratio of CSF Aβ_1–42_ to Aβ_1–40_ may be a more robust biomarker than CSF Aβ_1–42_ alone. These results contribute to our understanding of the key preanalytical factors impacting the measurement accuracy of Aβ peptides Aβ_1–42_, Aβ_1–40_, Aβ_1–38_, Aβ_1–37_, and Aβ_1–34_ and will contribute to emerging standardized operating procedures for handling CSF in clinical settings.

## Additional file

Additional file 1: **Table S1**. Tube Types **Table S2**. MRM transitions, cone voltages, and collision energies for each peptide and internal standards **Figure S1**. Impact of aliquot size on CSF Aβ1-42 concentrations measured from low-retention tubes with the MSD Aβ1-42 assay. Matched 0.1mL and 0.5-mL aliquots of CSF from AD patients (n=15) were measured. Aβ1-42 concentrations from 0.5-mL aliquots were higher than 0.1-mL aliquots in 15/15 subjects. **Figure S2**. GuHCl denaturation increases Aβ1-42 recovery from the CSF of YNC and AD subjects. (A) Aβ1-42 concentrations recovered from aliquots of YNC and AD subjects when the samples were denatured with GuHCl before transfer from the original aliquot tube. (B) Aβ1-42 concentrations recovered from aliquots of YNC and AD subjects when the samples were denatured with GuHCl after transfer from the original aliquot tube. **Figure S3**. Receiver Operating Characteristic (ROC) Curves generated for CSF Aβ1-42. ROC curves generated from YNC and AD CSF denatured with GuHCl before transfer from the original aliquot tube (black) or from the same samples denatured with GuHCl treatment after transfer from the original aliquot tube (gray).**Figure S4.** CSF Aβ peptide recovery after GuHCl denaturation. Recovery of Aβ1-42, Aβ1-40, and Aβ1-38, Aβ1-37, and Aβ1-34 from healthy controls (black) and Dementia patients (red) after GuHCl denaturation at different times following collection. Condition 1- GuHCl denaturation immediately after CSF collection; Condition 2- GuHCl denaturation immediately before sub-aliquoting; Condition 3- GuHCl denaturation after sub-aliquoting, and immediately before analysis; Condition 4- sub-aliquot into Eppendorf LoBind® tubes immediately after collection, GuHCl denaturation immediately before analysis. **Figure S5**. Normalization of CSF Aβ1-42 to Aβ1-40, Aβ1-38, or Aβ1-37 reduces variability in CSF Aβ1-42 recovery from Eppendorf LoBind® tubes.Variation in CSF Aβ1-42 recovery due to the timing of GuHCl denaturation (recovery expressed as intra-subject %CV of the mean recovery across all tested GuHCl denaturation workflows (Conditions 1-4, Figure 6) was reduced in 10/10 Young Normal Controls (YNC, black triangles) and 9/10 patients diagnosed with dementia (red circles) when the Aβ1-42 was normalized to Aβ1-40, Aβ1-38 or Aβ1-37 (****p≤ 0.0001).(DOCX 5078 kb)

## Abbreviations

2D-UPLC-MS/MS: Two-dimensional ultraperformance liquid chromatography-tandem mass spectrometry
aa: Amino acids
Aβ_1–42_: Amyloid-β 1–42
ACN: Acetonitrile
aCSF: Artificial cerebrospinal fluid
AD: Alzheimer’s disease
CSF: Cerebrospinal fluid
CV: Coefficient of variation
DEM: Dementia of unknown origin
ECL: Electrochemiluminescence
GuHCl: Guanidine hydrochloride
mAb: Monoclonal capture antibodies
MRM: Multiple-reaction monitoring
PET: Positron emission tomography
pTau181: Phosphorylated Tau181
YNC: Young, cognitively normal control subjects
