# Supplementary material for: Preanalytical approaches to improve recovery of amyloid-β peptides from CSF as measured by immunological or mass spectrometry-based assays
Source: Alzheimers Res Ther. 2018 Nov 28;10:118. doi: 10.1186/s13195-018-0445-0 (PMC6264029; doi:10.1186/s13195-018-0445-0)
Supplement: Supplementary file 1 — Table S1. Tube Types Table S2. MRM transitions, cone voltages, and collision energies for each peptide and internal standards Figure S1. Impact of aliquot size on CSF Aβ1-42 concentrations measured from low-retention tubes with the MSD Aβ1-42 assay. Matched 0.1mL and 0.5-mL aliquots of CSF from AD patients (n=15) were measured. Aβ1-42 concentrations from 0.5-mL aliquots were higher than 0.1-mL aliquots in 15/15 subjects. Figure S2. GuHCl denaturation increases Aβ1-42 recovery from the CSF of YNC and AD subjects. (A) Aβ1-42 concentrations recovered from aliquots of YNC and AD subjects when the samples were denatured with GuHCl before transfer from the original aliquot tube. (B) Aβ1-42 concentrations recovered from aliquots of YNC and AD subjects when the samples were denatured with GuHCl after transfer from the original aliquot tube. Figure S3. Receiver Operating Characteristic (ROC) Curves generated for CSF Aβ1-42. ROC curves generated from YNC and AD CSF denatured with GuHCl before transfer from the original aliquot tube (black) or from the same samples denatured with GuHCl treatment after transfer from the original aliquot tube (gray).Figure S4. CSF Aβ peptide recovery after GuHCl denaturation. Recovery of Aβ1-42, Aβ1-40, and Aβ1-38, Aβ1-37, and Aβ1-34 from healthy controls (black) and Dementia patients (red) after GuHCl denaturation at different times following collection. Condition 1- GuHCl denaturation immediately after CSF collection; Condition 2- GuHCl denaturation immediately before sub-aliquoting; Condition 3- GuHCl denaturation after sub-aliquoting, and immediately before analysis; Condition 4- sub-aliquot into Eppendorf LoBind® tubes immediately after collection, GuHCl denaturation immediately before analysis. Figure S5. Normalization of CSF Aβ1-42 to Aβ1-40, Aβ1-38, or Aβ1-37 reduces variability in CSF Aβ1-42 recovery from Eppendorf LoBind® tubes.Variation in CSF Aβ1-42 recovery due to the timing of GuHCl denaturation (recovery expressed as intra-subject [file 13195_2018_445_MOESM1_ESM.docx]

**Additional file 1**

**
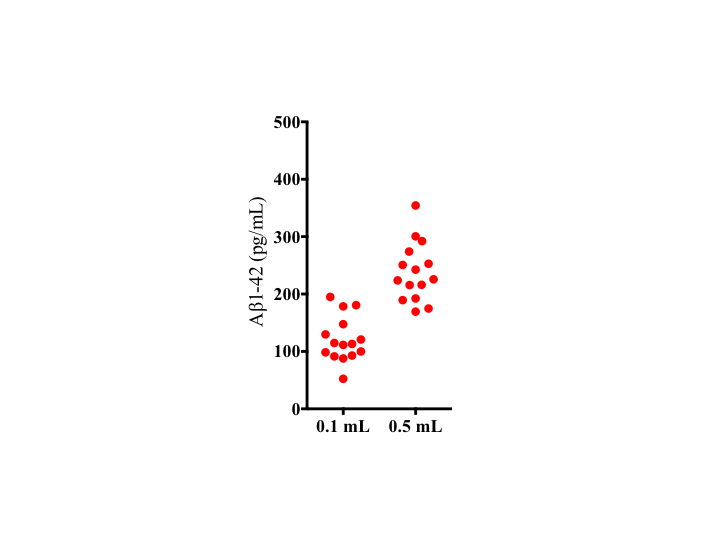
**

**Figure S1 Impact of aliquot size on CSF Aβ1-42 concentrations measured from low-retention tubes with an electrochemiluminescence Aβ1-42 assay.** Matched 0.1mL and 0.5-mL aliquots of CSF from AD patients (n=15) were measured with an electrochemiluminescence immunoassay specific for Aβ1-42. Aβ1-42 concentrations from 0.5-mL aliquots were higher than 0.1-mL aliquots in 15/15 subjects.

**
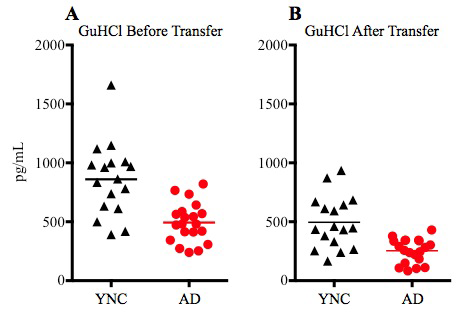
**

**Figure S2 GuHCl denaturation increases Aβ1-42 recovery from the CSF of YNC and AD subjects.**  (A) Aβ1-42 concentrations recovered from aliquots of YNC and AD subjects when the samples were denatured with GuHCl before transfer from the original aliquot tube. (B) Aβ1-42 concentrations recovered from aliquots of YNC and AD subjects when the samples were denatured with GuHCl after transfer from the original aliquot tube.

**
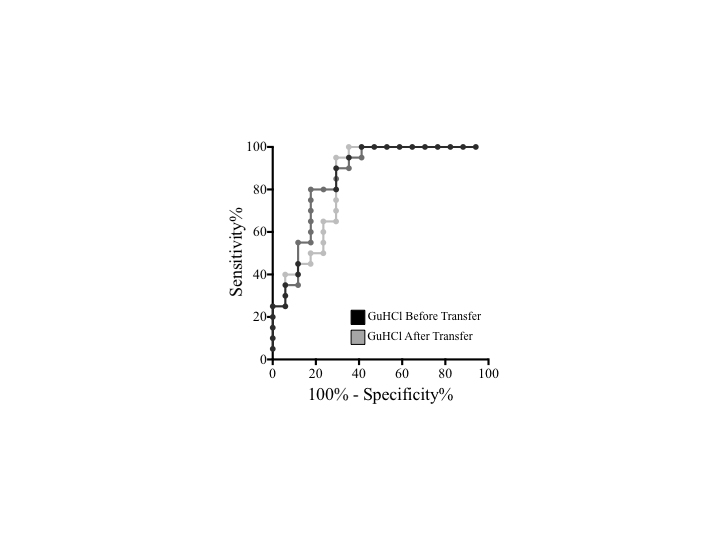
**

**Figure S3 Receiver Operating Characteristic (ROC) Curves generated for CSF Aβ1-42.** ROC curves generated from YNC and AD CSF denatured with GuHCl before transfer from the original aliquot tube (black) or from the same samples denatured with GuHCl treatment after transfer from the original aliquot tube (gray).


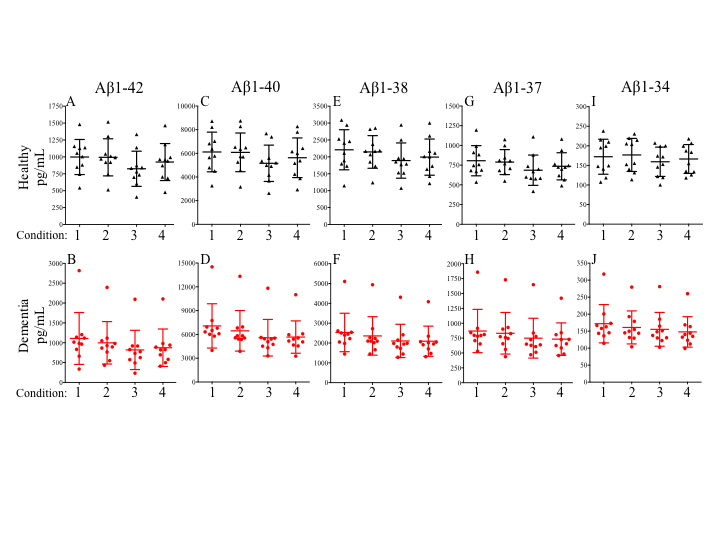


**Figure S4 CSF Aβ peptide recovery after GuHCl denaturation.** Recovery of Aβ1-42, Aβ1-40, and Aβ1-38, Aβ1-37, and Aβ1-34 from healthy controls (black) and Dementia patients (red) after GuHCl denaturation at different times following collection. Condition 1- GuHCl denaturation immediately after CSF collection; Condition 2- GuHCl denaturation immediately before sub-aliquoting; Condition 3- GuHCl denaturation after sub-aliquoting, and immediately before analysis; Condition 4- sub-aliquot into Eppendorf LoBind® tubes immediately after collection, GuHCl denaturation immediately before analysis.

**
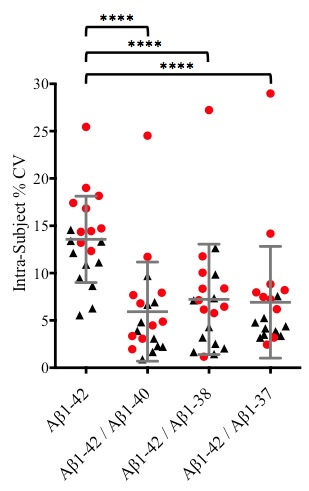
**

**S5 Normalization of CSF Aβ1-42 to Aβ1-40, Aβ1-38, or Aβ1-37 reduces variability in CSF Aβ1-42 recovery from Eppendorf LoBind® tubes.**

Variation in CSF Aβ1-42 recovery due to the timing of GuHCl denaturation (recovery expressed as intra-subject %CV of the mean recovery across all tested GuHCl denaturation workflows (Conditions 1-4, Figure 6)) was reduced in 10/10 Young Normal Controls (YNC, black triangles) and 9/10 patients diagnosed with dementia (red circles) when the Aβ1-42 was normalized to Aβ1-40, Aβ1-38 or Aβ1-37 (****p≤ 0.0001).
